# Ultrasonographic Synovitis Is Associated with the Development of Joint Destruction in Patients with Psoriatic Arthritis

**DOI:** 10.3390/jpm14060630

**Published:** 2024-06-13

**Authors:** Yutaro Yamada, Kentaro Inui, Koji Mandai, Kenji Mamoto, Tatsuya Koike, Chiharu Tateishi, Daisuke Tsuruta, Tadashi Okano

**Affiliations:** 1 Department of Orthopaedic Surgery, Osaka Metropolitan University Graduate School of Medicine, Osaka 545-8585, Japan; d21854y@omu.ac.jp (Y.Y.); k_inui2@nakatsu.saiseikai.or.jp (K.I.); j21198f@omu.ac.jp (K.M.); 2 Department of Orthopaedic Surgery, Osaka Saiseikai Nakatsu Hospital, Osaka 530-0012, Japan; 3 Mikunigaoka Mandai Orthopaedic Clinic, Osaka 590-0024, Japan; mandaiseikei@gmail.com; 4 Search Institute for Bone and Arthritis Disease (SINBAD), Wakayama 649-2211, Japan; tatsuya@omu.ac.jp; 5 Department of Dermatology, Osaka Metropolitan University Graduate School of Medicine, Osaka 545-8585, Japan; chiharu0326@omu.ac.jp (C.T.); c21328t@omu.ac.jp (D.T.); 6 Center for Senile Degenerative Disorders (CSDD), Osaka Metropolitan University Graduate School of Medicine, Osaka 545-8585, Japan

**Keywords:** psoriatic arthritis, ultrasound, synovitis, enthesitis

## Abstract

Background: Psoriatic arthritis (PsA) is characterized by enthesitis. As persistent inflammation around joints results in bone and cartilage destruction and physical impairment, a detailed assessment of inflammation is essential. We previously reported the difference between clinical assessment (tenderness) and ultrasound (US) assessment (inflammation) of entheses. Herein, we investigated whether clinical or US assessment of joints and entheses can predict the progression of joint destruction in Japanese patients with PsA. Methods: Thirty joints and 14 entheses in 47 patients were assessed using US and clinical examination. The US greyscale (GS) and power Doppler (PD) scores at the ultrasonographic synovitis, the US active enthesitis count, and the clinical tender joint/entheses count were assessed. Additionally, the yearly radiographic progression of the Sharp–van der Heijde scoring method for PsA was assessed. Their correlations were investigated. Results: About half of the patients with PsA experienced joint destruction during a follow-up period of 20.4 months. Progression of joint destruction in patients with PsA only correlated with joint GS and PD scores, reflecting the severity of ultrasonographic synovitis, not with the tender joint/entheses count. Conclusions: US examinations are essential for preventing joint destruction and physical impairment in patients with PsA.

## 1. Introduction

About 30–50% of patients with psoriasis are diagnosed as having psoriatic arthritis (PsA) because of coexistent clinical musculoskeletal manifestations [1,2,3]. PsA is characterized by enthesitis, which is the inflammation of the entheses located on tendons or ligament insertion to a bone [4]. Additionally, inflammation of a joint (arthritis), finger (dactylitis), and spine (spondylitis) are common manifestations of enthesitis.

PsA is usually treated according to the recommendations of the Group for Research and Assessment of Psoriasis and Psoriatic Arthritis (GRAPPA) or the European League Against Rheumatism (EULAR) [5,6]. In these recommendations, treatment should be enhanced according to the disease activity in the skin, entheses, joints, etc. In particular, patients with polyarthritis or prolonged mono/oligoarthritis should start methotrexate (MTX), while patients with enthesitis should start non-steroidal anti-inflammatory drugs (NSAIDs). If the inflammation has not improved, the administration of biological disease-modifying antirheumatic drugs (bDMARDs) should be considered. In order not to miss the opportunity to have enhanced treatment, assessment of enthesitis and arthritis is important and should be precise, as these inflammatory conditions are associated with disease activity, joint destruction, and functional impairment in PsA. Conventionally, enthesitis and arthritis are assessed by clinical examination that evaluates the presence of tenderness in entheses and joints, but this does not reflect an inflammatory condition. There are limitations in the reliability, validity, and sensitivity of clinical assessment, such as the Leeds Enthesitis Index or Tender Joint Count [7,8,9]. As we previously reported, an inflammatory condition is different from the presence of tenderness in PsA, such as rheumatoid arthritis [10]. If inflammation persists without tenderness (subclinical inflammation) in entheses or joints, joint destruction may occur by missing the opportunity to have enhanced treatment.

Ultrasound (US) is a relatively feasible and sensitive method of precisely assessing inflammatory conditions in entheses and joints. US examinations may be more sensitive and useful than clinical evaluations in detecting subclinical inflammation. US adds sensitivity and specificity to sites of disease in PsA. Several recent studies have revealed the value of US in improving the understanding of the disease [11,12,13]. In fact, the Outcome Measures in Rheumatology (OMERACT) US Specialist Interest Group reached an agreement regarding US assessment definitions [14].

Although US examination may be essential to assess the present disease activity and the potential for subsequent joint destruction and physical impairment, clinical assessment such as tenderness at entheses is still in frequent use in many clinical trials in PsA. Herein, we conducted the study to confirm the importance of US examination when assessing the disease condition of PsA. We investigated the relationships with clinical features, including disease activity and joint destruction by comparing US and clinical assessments of entheses and joints for the presence of inflammation in Japanese patients with PsA. As there was no correlation between joint destruction and any clinical features including US and clinical detection of enthesitis or arthritis, we conducted a follow-up study because the progression of joint destruction should be longitudinally associated with inflammatory conditions. Thus, this follow-up study aimed to investigate the longitudinal relationships between US examination and clinical assessments of entheses and joints and joint destruction (structural damage) in patients with PsA.

## 2. Materials and Methods

### 2.1. Patients

A total of 107 patients with psoriasis treated at the Department of Dermatology in our institution were referred to the Department of Orthopedics because of any suspicious musculoskeletal manifestations from January 2015 to March 2017. Among these, 63 patients fulfilled the CASPAR (ClASsification criteria for Psoriatic Arthritis) and were diagnosed as PsA. Finally, 47 patients without missing data agreed to enroll in this study. A detailed setting was described previously [10]. The study was registered as the ISLAND study (Identification of riSk factors for spondyLoArthropathy in patieNts Diagnosed with psoriasis), which is a cohort study of PsA patients, in the University Hospital Medical Information Network (UMIN) Clinical Trials Registry (registration number: UMIN000024292). The Osaka Metropolitan University Hospital Certified Review Board approved the study protocol, which was conducted in accordance with the Declaration of Helsinki (approval number: 3146). Informed consent for participation in this study was provided by all the patients.

### 2.2. Study Measures

Patient characteristics, including age, disease durations of psoriasis, and musculoskeletal manifestations, were recorded at baseline. Surveys, including the health assessment questionnaire (HAQ), as a measure of functional status, and the Psoriatic Arthritis Screening and Evaluation (PASE), as a measure of musculoskeletal involvement, were completed. The Psoriasis Area Severity Index (PASI) was calculated as a measure of skin disease severity assessed by dermatologists, and the Disease Activity in Psoriatic Arthritis (DAPSA) (26) and Disease Activity Score 28 with C-reactive protein (DAS28-CRP) were calculated as measures of disease severity in the peripheral joints or entheses. The patients underwent blood examinations for CRP and matrix metalloproteinase-3 levels (MMP-3). 

Radiographic examinations of bilateral hands, feet, sacroiliac joints, and the entire spine at baseline and follow-up (20.4 months after baseline) were performed. The Sharp–van der Heijde scoring method for PsA (SvdH), as a measure of joint destruction, was calculated at baseline and follow-up [15]. Finally, ΔSvdH/year was calculated, as the yearly progression in bone structural damage. When ΔSvdH/year was >0.5, it was considered as “joint destruction.”

### 2.3. US and Clinical Assessments of Joints and Enthesitis

A total of 34 sites in 30 joints, including bilateral wrists (radial, middle, and ulnar side), proximal/distal interphalangeal, and metacarpophalangeal joints in bilateral thumbs and fingers were examined by three expert sonographers certified by the Japan College of Rheumatology using a HI-VISION ASCENDUS US system (Hitachi-Aloka Medical, Tokyo, Japan) with a 18MHz linear transducer. The evaluated 34 sites are included in the sites evaluated by SvDH. Additionally, 14 entheses, including the bilateral humeral lateral epicondyles and insertions of the triceps, distal quadriceps tendons, proximal/distal patella tendons, Achilles tendons, and plantar fascia, were examined. The 14 evaluated entheses are included in the Outcome Measures in Rheumatology (OMERACT) US definition and MASEI (MAdrid Sonographic Enthesis Index) score [14,16]. Greyscale (GS) imaging parameters were set to obtain maximal contrast between all the structures. PD settings were standardized to the following values: pulse repetition frequency, 800 Hz; and Doppler frequency, 7.5 or 10 MHz. The color gain was set just below the level at which color noise appeared at the underlying bone.

Each joint was semi-quantitatively scored from 0–3 (0 = no synovial hypertrophy, 3 = severe synovial hypertrophy) in GS and 0–3 (0 = normal vascularity, 3 = severe hyperemia indicating inflammation) in PD. The sum of the 34 sites was calculated using the GS and PD scores (score, 0–102). Higher GS and PD scores indicate “severe arthritis”. Each joint was examined with a 0-degree extension of the thumb and fingers while g the hands were on a table.

Similarly, we counted the entheses presenting as “US active enthesitis”, which were evaluated by the inflammatory components of the OMERACT definition (positive PD signals with hypoechoic and/or thickened insertion of the tendon within 2 mm from the bone surface) at the 14 entheses. As the PD signal is usually difficult to find at the plantar fascia, owing to its depth and the thick skin of the plantar, we evaluated the presence of thickening at this site, rather than the PD signal. The bilateral humeral lateral epicondyles and triceps insertions to the olecranon were examined in the seated position, with the elbow at 90 degrees flexion and the forearm in the neutral position. The distal quadriceps and proximal/distal patella tendons were examined in the supine position, with the knee at 30 degrees flexion. The Achilles tendon and plantar fascia were examined in the prone position. We did not assess the presence of enthesophytes, as it is common in post-traumatic conditions and in those with degenerative changes.

Additionally, we evaluated the 30 joints and 14 entheses by clinical assessment. When patients felt tenderness at these sites, it was considered a tender joint or enthesis. When there was swelling in each of the 30 joints, it was considered a swollen joint. Tender and/or swollen joints and tender entheses counts were calculated. 

### 2.4. Evaluation of the Relationships between US/Clinical Arthritis and Enthesitis and Progression in Joint Destruction

Tender and/or swollen joints and tender entheses counts, US active enthesitis count, and severity of arthritis on US (sum of GS and PD scores) were finally evaluated. In other words, the severity of peripheral musculoskeletal manifestations at 30 joints and 14 entheses were semi-quantitatively scored by US and clinical assessment. The relationship between clinical features, including inflammatory markers and measures of disease activity and yearly progression in joint destruction, was investigated to identify which score is the best predictive factor for joint destruction.

### 2.5. Statistical Analysis

Patient characteristics are displayed as means ± standard deviations. Associations between the arthritis/enthesitis and clinical features, including progression in joint destruction, were evaluated using Spearman’s correlation coefficient. Multivariate analysis of predictive factors for joint destruction in patients with PsA was performed using multiple regression analysis. All statistical analyses were performed using EZR ver1.37 [17]. All *p* values were two-sided, and *p* < 0.05 was considered statistically significant.

## 3. Results

### 3.1. Patients’ Characteristics

The characteristics of the 47 patients with PsA are shown in Table 1. Skin symptoms were relatively well-controlled (mean PASI was 7.2 and DASPA was 20.4). The disease activity in peripheral joints was moderate (mean DAS28-CRP was 3.23). Some patients were treated with NSAIDs (14.9%), MTX (19.1%) for musculoskeletal symptoms, and/or bDMARDs (14.9%; infliximab, *n* = 3; adalimumab, *n* = 3; ustekinumab, *n* = 1) for skin lesions. The mean SvdH was 12.6 points at baseline.

### 3.2. Prevalence of US/Clinical Arthritis and Enthesitis

US finding of active enthesitis was more prevalent (79%) than clinical entheseal tenderness (49%) in the 14 entheses of 47 patients, and there was no concordance. A mean US active enthesitis count was 3.09, and a tender entheses count was 1.72 (of the 14 entheses). 

As for arthritis, joint GS and PD scores were 5.00 and 2.74, respectively. In contrast, the mean tender/swollen joint count was 5.93/2.56 of 30 joints. Patients with GS or PD positive scores were more prevalent (70.2%) than patients with any swollen joint (46.8%).

### 3.3. Prevalence and Predictive Factor for Progression in Joint Destruction

**Figure 1:**
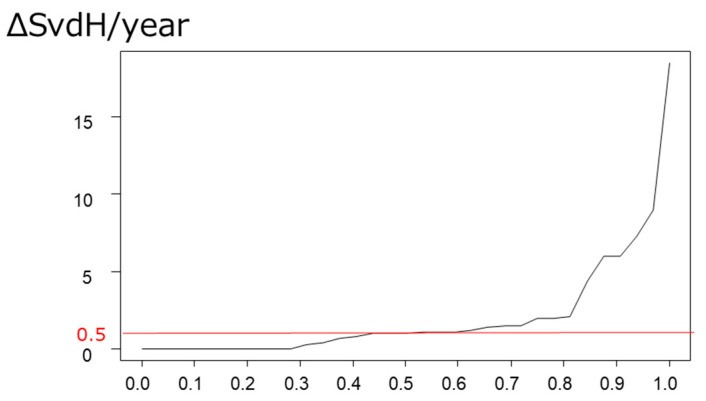
Cumulative probability plot of joint destruction. The mean ΔSvdH/year was 2.12, and 56.3% had joint destruction (ΔSvdH/year > 0.5). SvdH, Sharp–van der Heijde scoring method for PsA.

The mean ΔSvdH/year was 2.12, and 56.3% of the 47 patients had joint destruction (ΔSvdH/year > 0.5) during a follow-up period of 20.4 months from baseline. Cumulative probability plots of joint destruction are shown in Figure 1.

Univariate analysis of the predictive factors for joint destruction (ΔSvdH/year) in patients with PsA is shown in Table 2. Swollen joint count (r = 0.13, *p* = 0.48), tender joint count (r = −0.10, *p* = 0.58), and tender entheses count (r = −0.19, *p* = 0.30) did not correlate with joint destruction. The US active enthesitis count did not correlate with joint destruction (r = −0.13, *p* = 0.48), while GS and PD scores for US assessment of arthritis significantly correlated with joint destruction (r = 0.44, *p* = 0.01; r = 0.38, *p* = 0.03, respectively). Additionally, joint destruction significantly correlated with age (r = 0.44, *p* = 0.01) and MTX dose (r = 0.38, *p* = 0.03). Disease activity of the skin (PASI), musculoskeletal involvement (PASE, DAS28CRP, and DAPSA), physical function (HAQ), and inflammatory markers (CRP, MMP-3) did not correlate with joint destruction. 

The multivariate regression analysis showed that the GS score was a significant predictive factor for joint destruction (β = 0.45, *p* < 0.001) when GS score and age were used as explanatory variables (Table 2).

## 4. Discussion

We assessed 30 joints and 14 entheses using US and conventional clinical assessments; additionally, we investigated their association with progression to joint destruction in 47 patients with PsA. We found that the progression of joint destruction only correlated with GS and PD scores, which is a measure of arthritis detected by US. The joint destruction did not correlate with conventional clinical assessment including tenderness at the entheses or tender/swollen joint counts in the longitudinal analysis. These facts support previous reports regarding the difference between US and clinical assessments and emphasize the importance of US when assessing inflammation precisely.

Brown et al. reported that joint destruction correlated with the joint PD score and emphasized the use of US imaging for the accurate evaluation of disease activity and prediction of joint destruction in rheumatoid arthritis [18]. Similarly, US assessment is also important in the prediction of joint destruction in PsA; however, this is not frequently reported in clinical trials. We previously reported that US assessment detected enthesitis more frequently than clinical assessment and that there was no concordance between US active enthesitis and clinical tenderness [10]. This fact suggests that some patients have subclinical inflammation, which may result in joint destruction and physical impairment. However, there was no correlation between joint destruction and US active enthesitis count, clinical tenderness count, disease activity, or CRP cross-sectionally at baseline. We hypothesized that US detection of enthesitis or arthritis is longitudinally associated with joint destruction and conducted a follow-up study.

In the present study, joint destruction correlated with joint GS and PD scores, but not with the US active enthesitis count. This is somewhat different from a previous report in which both the joint PD score and the presence of PD at entheses, as assessed by US, are the predictors of progression to joint destruction in PsA [19]. These differences are probably because the US active enthesitis count assessed in this study does not reflect the severity of inflammation, whereas the previous report used the Glasgow Ultrasound Enthesitis Scoring System (GUESS), which assesses the severity of inflammation at the entheses [20,21]. In fact, the joint PD score, which reflects the severity of inflammation, correlated with joint destruction. We assessed only the presence or absence of enthesitis, as defined by the OMERACT US initiative. Another reason may be the relatively small number of samples in this study.

There are different methods of assessing joint destruction in patients with PsA. Compared to US and magnetic resonance imaging (MRI), an X-ray is quick and feasible, but it lacks sensitivity for detecting early destructive change due to inflammation in joints and entheses. Furthermore, US has advantages over an MRI in accessibility, cost, and lack of contraindications. US can detect subclinical synovitis and enthesitis, that is present inflammation, despite the absence of clinical tenderness. Subclinical synovitis and enthesitis may cause joint destruction and physical impairment. A diagnostic delay may result in irreversible joint damage, disability, and a shorter survival period. Thus, it is important not to miss such subclinical inflammation. US can detect inflammatory changes, even in the early stage, thereby enabling early intervention [11]. Furthermore, US assessments of enthesitis are reported for high reliability [22]. Thus, US is useful for assessing enthesitis and early diagnosis of PsA. The OMERACT US initiative reached an agreement regarding US assessment definitions; it was concluded that a positive PD signal and the presence of enthesophytes are the most reliable and feasible US findings of enthesitis [15].

As we previously reported, tenderness is completely different from inflammation, which cannot be assessed only by tenderness in patients with PsA or rheumatoid arthritis. Thus, an inflammatory condition should be assessed by an US examination, and not by tenderness.

When inflammation is present at entheses and/or joints, it is recommended to administer NSAIDs and/or DMARDs depending on the sites of inflammation, according to GRAPPA or EULAR recommendations. Especially, the patients with polyarthritis should have MTX unless contraindicated. If the inflammatory condition has not improved after the treatment, it should be enhanced (e.g., bDMARDs administration) without missing the opportunity. Joint destruction or physical impairment can be prevented by these algorithms for the management of PsA. However, the efficacy of MTX or bDMARDs for preventing joint destruction is unknown in the present study, as this is just an observational study. The MTX dose was correlated with the development of joint destruction. The reason is probably because some patients with severe arthritis (high GS/PD scores in US), which may result in joint destruction, were already treated with MTX at baseline.

The present study has several limitations. First, US examination of foot joints, assessed by SvdH, was not conducted; therefore, we cannot perfectly support the correlation between the severity of arthritis and joint destruction, although the assessed sites in this study were similar to the previous reports. Second, the US assessment may have overestimated enthesitis because positive PD signals at the entheses were also observed in conditions of overuse. Finally, the present study comprised a small number of patients.

In conclusion, joint destruction in patients with PsA only correlated with GS and PD scores, thereby reflecting the severity of ultrasonographic synovitis. These results suggest that US examinations are essential in preventing joint destruction and physical impairment in patients with PsA.

## Figures and Tables

**Table 1:** Baseline characteristics of all patients with PsA.

Patients with PsA (*n* = 47)	
Age, years	56.4 ± 15.2
Female, %	53.2
BMI, kg/m^2^	23.6 ± 4.1
Disease duration for psoriasis, months	169.9 ± 164.1
Disease duration for PsA, months	90.8 ± 123.6
NSAIDs use rate, %	14.9
MTX use rate, %	19.1
bDMARDs use rate, %	14.9
PASE	45.9 ± 15.2
PASI	7.2 ± 10.1
DAPSA	20.4 ± 18.2
DAS28-CRP	3.23 ± 1.37
mHAQ	0.47 ± 0.48
SvdH	12.6 ± 18.6
CRP, mg/dL	0.90 ± 2.46
MMP-3, ng/mL	84.35 ± 53.91
Tender entheses count	1.72 ± 2.62
US active enthesitis count	3.09 ± 2.55
Tender joint count	5.93 ± 5.92
Swollen joint count	2.56 ± 3.92
US arthritis assessment—GS score US arthritis assessment—PD score	5.00 ± 4.55 2.74 ± 3.74

Data are shown as mean ± standard deviation. PsA, psoriatic arthritis; BMI, body mass index, NSAIDs, non-steroidal anti-inflammatory drugs; MTX, methotrexate; bDMARDs, biological disease-modifying antirheumatic drugs; PASE, Psoriatic Arthritis Screening and Evaluation, PASI, Psoriasis Area Severity Index; DAPSA, Disease Activity in Psoriatic Arthritis; DAS, Disease Activity Score; CRP, C-reactive protein; mHAQ, modified Health Assessment Questionnaire; SvdH, Sharp–van der Heijde scoring method for PsA; MMP-3, matrix metalloproteinase 3; GS, grayscale; PD, power Doppler.

**Table 2:** Univariate and multivariate regression analysis of predictive factors for progression of joint damage in patients with PsA.

	Univariate		Multivariate	
	R-Value	*p* Value	β Value	*p* Value
Age	0.44	0.01	0.04	0.13
PASE	0.12	0.52	-	-
PASI	−0.01	0.96	-	-
DAS28CRP	0.07	0.71	-	-
DAPSA	−0.01	0.97	-	-
HAQ	−0.07	0.73	-	-
CRP	0.23	0.20	-	-
MMP-3	0.29	0.12	-	-
MTX dose	0.38	0.03	-	-
bDMARDs use	−0.11	0.54	-	-
Tender entheses count	−0.19	0.30	-	-
US active enthesitis counts	−0.13	0.48	-	-
Tender joint count	−0.10	0.58	-	-
Swollen joint count	0.13	0.48	-	-
Joint GS score	0.44	0.01	0.45	<0.001
Joint PD score	0.38	0.03	-	-

PsA, psoriatic arthritis; PASE, Psoriatic Arthritis Screening and Evaluation; PASI, Psoriasis Area Severity Index; DAPSA, Disease Activity in Psoriatic Arthritis; DAS, Disease Activity Score; CRP, C-reactive protein, HAQ; Health Assessment Questionnaire; MMP-3, matrix metalloproteinase 3; MTX, methotrexate; GS, gray scale; PD, power Doppler.

## Data Availability

The raw data supporting the conclusions of this article will be made available by the authors on request.

## References

[1] Ohara Y., Kishimoto M., Takizawa N., Yoshida K., Okada M., Eto H., Deshpande G.A., Ritchlin C.T., Tanaka A., Higashiyama M. (2015). Prevalence and Clinical Characteristics of Psoriatic Arthritis in Japan. J. Rheumatol..

[2] Yamamoto T., Ohtsuki M., Sano S., Igarashi A., Morita A., Okuyama R., Kawada A., Working Group of the Epidemiological Survey in the Japanese Society for Psoriasis Research (2016). Epidemiological analysis of psoriatic arthritis patients in Japan. J. Dermatol..

[3] Gladman D.D., Chandran V. (2011). Observational cohort studies: Lessons learnt from the University of Toronto Psoriatic Arthritis Program. Rheumatology.

[4] Azuaga A.B., Ramírez J., Cañete J.D. (2023). Psoriatic Arthritis: Pathogenesis and Targeted Therapies. Int. J. Mol. Sci..

[5] Gossec L., Smolen J.S., Ramiro S., de Wit M., Cutolo M., Dougados M., Emery P., Landewe R., Oliver S., Aletaha D. (2024). European League Against Rheumatism (EULAR) recommendations for the management of psoriatic arthritis with pharmacological therapies: 2015 update. Ann. Rheum. Dis..

[6] Coates L.C., Kavanaugh A., Mease P.J., Soriano E.R., Laura Acosta-Felquer M., Armstrong A.W., Bautista-Molano W., Boehncke W.H., Campbell W., Cauli A. (2016). Group for Research and Assessment of Psoriasis and Psoriatic Arthritis 2015 Treatment Recommendations for Psoriatic Arthritis. Arthritis Rheumatol..

[7] Mease P.J. (2011). Measures of psoriatic arthritis: Tender and Swollen Joint Assessment, Psoriasis Area and Severity Index (PASI), Nail Psoriasis Severity Index (NAPSI), Modified Nail Psoriasis Severity Index (mNAPSI), Mander/Newcastle Enthesitis Index (MEI), Leeds Enthesitis Index (LEI), Spondyloarthritis Research Consortium of Canada (SPARCC), Maastricht Ankylosing Spondylitis Enthesis Score (MASES), Leeds Dactylitis Index (LDI), Patient Global for Psoriatic Arthritis, Dermatology Life Quality Index (DLQI), Psoriatic Arthritis Quality of Life (PsAQOL), Functional Assessment of Chronic Illness Therapy-Fatigue (FACIT-F), Psoriatic Arthritis Response Criteria (PsARC), Psoriatic Arthritis Joint Activity Index (PsAJAI), Disease Activity in Psoriatic Arthritis (DAPSA), and Composite Psoriatic Disease Activity Index (CPDAI). Arthritis Care Res..

[8] Gandjbakhch F., Terslev L., Joshua F., Wakefield R.J., Naredo E., D’Agostino M.A. (2011). Ultrasound in the evaluation of enthesitis: Status and perspectives. Arthritis Res. Ther..

[9] Healy P.J., Helliwell P.S. (2008). Measuring clinical enthesitis in psoriatic arthritis: Assessment of existing measures and development of an instrument specific to psoriatic arthritis. Arthritis Rheumatol..

[10] Yamada Y., Inui K., Okano T., Mandai K., Mamoto K., Koike T., Takeda S., Yamashita E., Yoshida Y., Tateishi C. (2021). Ultrasound assessment, unlike clinical assessment, reflects enthesitis in patients with psoriatic arthritis. Clin. Exp. Rheumatol..

[11] Dubash S.R., De Marco G., Wakefield R.J., Tan A.L., McGonagle D., Marzo-Ortega H. (2020). Ultrasound Imaging in Psoriatic Arthritis: What Have We Learnt in the Last Five Years?. Front. Med..

[12] D’Agostino M.A. (2018). Enthesitis detection by ultrasound: Where are we now?. Clin. Exp. Rheumatol..

[13] Bandinelli F., Prignano F., Bonciani D., Bartoli F., Collaku L., Candelieri A., Lotti T., Matucci-Cerinic M. (2013). Ultrasound detects occult entheseal involvement in early psoriatic arthritis independently of clinical features and psoriasis severity. Clin. Exp. Rheumatol..

[14] Balint P.V., Terslev L., Aegerter P., Bruyn G.A.W., Chary-Valckenaere I., Gandjbakhch F., Iagnocco A., Jousse-Joulin S., Moller I., Naredo E. (2018). Reliability of a consensus-based ultrasound definition and scoring for enthesitis in spondyloarthritis and psoriatic arthritis: An OMERACT US initiative. Ann. Rheum. Dis..

[15] van der Heijde D., Gladman D.D., Kavanaugh A., Mease P.J. (2020). Assessing structural damage progression in psoriatic arthritis and its role as an outcome in research. Arthritis Res. Ther..

[16] de Miguel E., Cobo T., Munoz-Fernandez S., Naredo E., Uson J., Acebes J.C., Andreu J.L., Martin-Mola E. (2009). Validity of enthesis ultrasound assessment in spondyloarthropathy. Ann. Rheum. Dis..

[17] Kanda Y. (2013). Investigation of the freely available easy-to-use software ‘EZR’ for medical statistics. Bone Marrow Transplant..

[18] Brown A.K., Conaghan P.G., Karim Z., Quinn M.A., Ikeda K., Peterfy C.G., Hensor E., Wakefield R.J., O’Connor P.J., Emery P. (2008). An explanation for the apparent dissociation between clinical remission and continued structural deterioration in rheumatoid arthritis. Arthritis Rheumatol..

[19] El Miedany Y., El Gaafary M., Youssef S., Ahmed I., Nasr A. (2015). Tailored approach to early psoriatic arthritis patients: Clinical and ultrasonographic predictors for structural joint damage. Clin. Rheumatol..

[20] Elalouf O., Bakirci Ureyen S., Touma Z., Anderson M., Kaeley G.S., Aydin S.Z., Eder L. (2019). Psoriatic Arthritis Sonographic Enthesitis Instruments: A Systematic Review of the Literature. J. Rheumatol..

[21] Agache M., Popescu C.C., Popa L., Codreanu C. (2022). Ultrasound Enthesitis in Psoriasis Patients with or without Psoriatic Arthritis, a Cross-Sectional Analysis. Medicina.

[22] Bonfiglioli K.R., Lopes F.O.d.A., Figueiredo L.Q.d., Ferrari L.F.F., Guedes L. (2024). Ultrasonographic Insights into Peripheral Psoriatic Arthritis: Updates in Diagnosis and Monitoring. J. Pers. Med..

